# Supplementary material for: Predicting visual function by interpreting a neuronal wiring diagram
Source: Nature. 2024 Oct 2;634(8032):113–23. doi: 10.1038/s41586-024-07953-5 (PMC11446822; doi:10.1038/s41586-024-07953-5)
Supplement: Supplementary file 6 — CRF and ERF predictions for individual TmY4 and TmY9 cells. Analogous to Supplementary Data 3, but for TmY target types. Shown are the top four monosynaptic pathways, the strongest pathway passing through each of the top ten intermediary types (ranking from Extended Data Fig. 7), and the trisynaptic pathway Tm1–TmY–Dm3–TmY (see the section entitled Prediction of spatial normalization). [file 41586_2024_7953_MOESM6_ESM.zip › DataS4/TmY9qΓèÑ/720575940632285132.pdf]

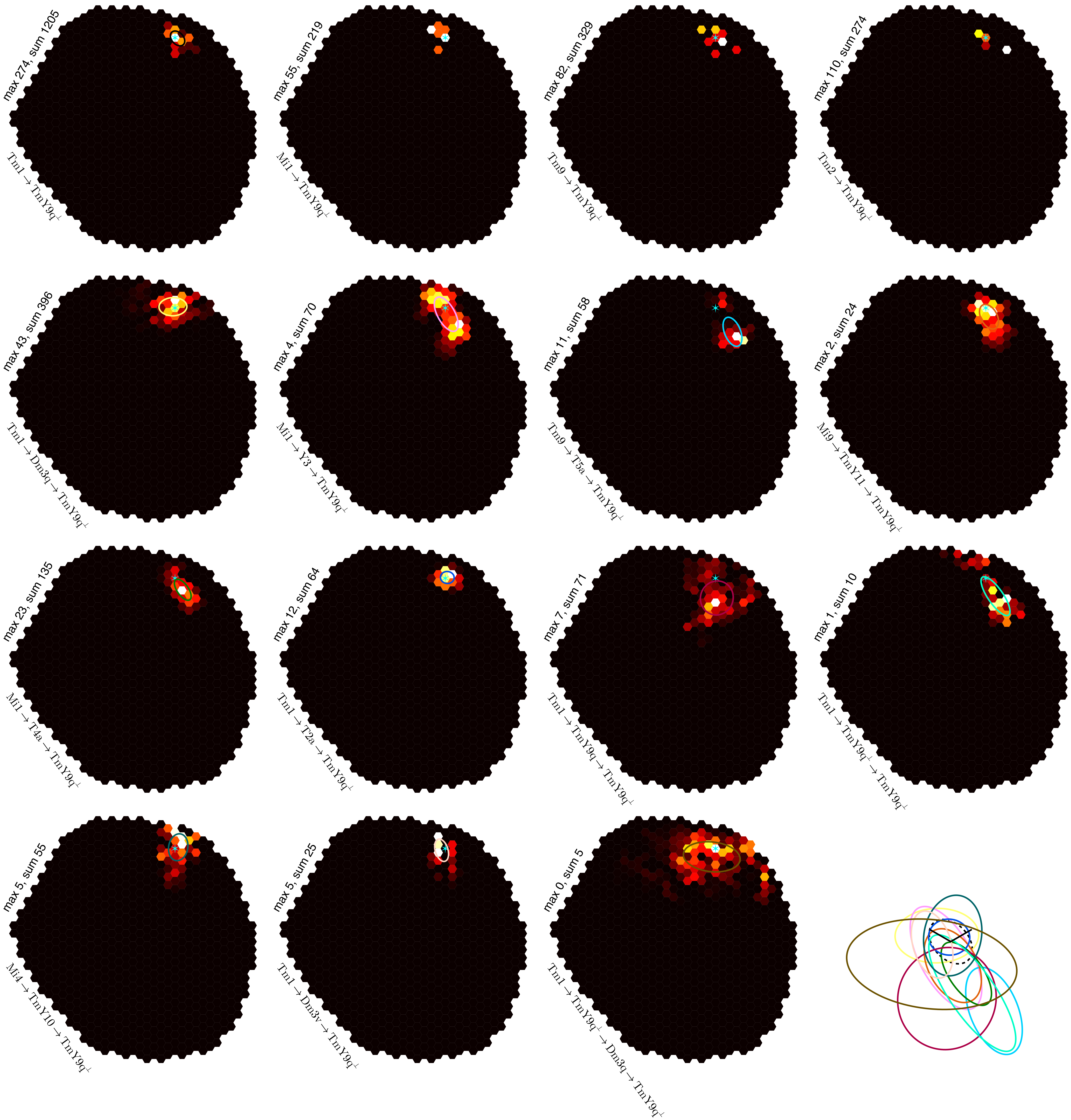
max 274, sum 1205
Tm1 → TmY9q⊥
max 55, sum 219
Mi1 → TmY9q⊥
max 82, sum 329
Tm9 → TmY9q⊥
max 110, sum 274
Tm2 → TmY9q⊥
max 43, sum 396
Tm1 → Dm3q → TmY9q⊥
max 4, sum 70
Mi1 → Y3 → TmY9q⊥
max 11, sum 58
Tm9 → T5a → TmY9q⊥
max 2, sum 24
Mi9 → TmY11 → TmY9q⊥
max 23, sum 135
Mi1 → T4a → TmY9q⊥
max 12, sum 64
Tm1 → T2a → TmY9q⊥
max 7, sum 71
Tm1 → TmY9q → TmY9q⊥
max 1, sum 10
Tm1 → TmY9q⊥ → TmY9q⊥
max 5, sum 55
Mi4 → TmY10 → TmY9q⊥
max 5, sum 25
Tm1 → Dm3v → TmY9q⊥
max 0, sum 5
Tm1 → TmY9q⊥ → Dm3q → TmY9q⊥
